# Adaptability factors and behavioral biases of investors in frontier markets: An adaptive market hypothesis perspective

**DOI:** 10.1371/journal.pone.0345883

**Published:** 2026-03-26

**Authors:** Jannatunnesa Jannatunnesa, Nurul Shahnaz Mahdzan, Abu Hanifa Md Noman

**Affiliations:** 1 UM Business School, Faculty of Business and Economics, Universiti Malaya, Kuala Lumpur, Malaysia; 2 Department of Finance, Faculty of Business and Economics, Universiti Malaya, Kuala Lumpur, Malaysia; 3 Faculty of Social Science, Southampton Malaysia Business School, University of Southampton-Malaysia Campus, Iskandar Puteri, Johor, Malaysia; AAFT University of Media and Arts, INDIA

## Abstract

The Adaptive Market Hypothesis (AMH) suggests that investors are imperfect but adaptive, allowing behavioral biases to persist and evolve over time. However, empirical research on how adaptability factors influence these biases in stock investment remains limited, especially in frontier markets. This study examines the impact of adaptability factors on herding (HRD) and overconfidence (OVR) biases among individual investors in Bangladesh, a frontier market. Employing purposive sampling, data were collected through structured face-to-face and online questionnaire surveys from 640 retail investors on the Dhaka Stock Exchange (DSE). Data were managed using the Statistical Package for the Social Sciences (SPSS) and analyzed using SmartPLS 4.0 for Partial Least Squares Structural Equation Modeling (PLS-SEM). The results show that all the external adaptability factors (*social influence, consultation with financial advisors,* and *media*) positively influence herding, while the internal adaptability factors (*trading experience, self-reflection,* and *desire for learning*) positively influence overconfidence. Financial literacy negatively affects both biases. The study offers insights for policymakers, regulators, and investors on the cognitive and social elements driving biased investment decisions, especially in frontier markets. To the best of the authors’ knowledge, this study pioneers a market adaptability model incorporating novel variables, labelled ‘adaptability factors’, grounded in AMH and bounded rationality.

## Introduction

Traditional finance theories assume that markets are efficient and that investors act rationally, maximizing utility through informed decisions [1,2]. Recent studies align with this, suggesting that investors make rational investment choices based on risk-return trade-offs [3]. In contrast, behavioral finance proposes that instead of pure rationality, investment decisions are shaped by psychological factors, especially in risky situations [4–6].

The Adaptive Market Hypothesis (AMH) [7–9] offers a more nuanced view of market efficiency stating that markets alternate between phases of efficiency and inefficiency over time [10]. Based on bounded rationality [11] and dynamic market efficiency, AMH posits that investors learn from mistakes and adapt for survival in changing conditions [8]. *Market dynamics,* coupled with both *internal and external factors,* significantly shape investor adaptability. Although AMH has gained attraction, a comprehensive examination of individual factors influencing behavioral rationality in time-varying efficient markets is lacking [12]. This study addresses this gap by introducing the concept of “adaptability factors”, categorized as internal (cognitive) and external (social) dimensions.

Heuristics-based decisions, or behavioral biases, often lead to investors’ irrationality [7,8,12]. If unaddressed, these biases threaten investors’ survival in dynamic markets and destabilize financial systems. While herding (HRD) and overconfidence (OVR) biases have been widely studied, their connections to investors’ cognitive and social adaptability remain underexplored in frontier markets. These biases have been especially destabilizing markets in the post-COVID-19 era, significantly contributing to volatility and price distortions [13–16]. However, the psychological and social drivers behind these behaviors are still not well understood. Examining these biases through the lens of adaptability factors provides a timely and relevant contribution to behavioral finance, particularly in underrepresented and volatile markets.

Behavioral biases in frontier markets remain underexplored despite their heightened susceptibility to irrational investor behavior due to low liquidity, weak regulation, and high volatility [12,17,18]. Bangladesh, still classified as a frontier market [19], presents a compelling case with around 80% retail participation, as stated by the regulators [20], and frequent episodes of herding and overconfidence [21,22]. Cultural collectivism fosters conformity and trend-following, while low financial literacy amplifies cognitive biases. Market disruptions, such as the 2010 and 2013 crash, illustrate how structural weaknesses and psychological factors drive irrational decisions [12]. Studying Bangladesh’s market through the lens of AMH provides context-specific insights into how adaptability factors influence investor behavior under dynamic and volatile settings.

Based on the preceding discussion, the objectives of this study are: (RO1) to examine the influence of adaptability factors on herd bias of Bangladeshi retail investors; and (RO2) to examine the influence of adaptability factors on overconfidence bias.

This study contributes to behavioral finance in three ways. *First,* it introduces the concept of ‘adaptability factors’, encompassing both internal and external elements essential for investors in competitive, evolving markets. *Second,* it empirically examines their impact on behavioral biases (HRD and OVR) in stock market investments, contributing to the under-explored literature on behavioral biases in adaptive financial markets. *Third,* it provides a contextual contribution by examining market adaptability factors and behavioral biases in Bangladesh – a frontier stock market where retail dominance and structural fragility provide a critical lens on investor adaptability.

The rest of this paper is organized as follows. *Section 2* reviews literature and develops research hypotheses. *Section 3* outlines the methodology. *Section 4* presents empirical analysis. *Section 5* discusses the findings and *Section 6* concludes.

## Literature review and hypothesis development

### Herd bias

Herding (HRD) refers to the tendency of investors to imitate others’ behavior rather than relying on their own information, especially in uncertain environments [23,24]. This behavior is particularly strong in frontier markets, where informational transparency is limited [17,25]. Herd behavior is evolutionary and may decline over time as investors learn from mistakes [26]. Prior studies indicate that trading experience, financial literacy, and a desire for learning can mitigate herding while promoting rational decision-making [27,28]. Conversely, social indications, such as word-of-mouth communication or peer influence can significantly intensify herding behavior [29]. Given its widespread destabilizing impact on financial systems [30], understanding the drivers of herding is vital for improving market behavior and investor decision-making.

### Overconfidence bias

Overconfidence (OVR), a well-known heuristic-driven bias, occurs when individuals overestimate their knowledge, the accuracy of information and their ability to predict outcomes [31,32]. Research identifies several antecedents of overconfidence, including demographics [33], trading experience [34], financial literacy [30], and social factors, such as, information from advisors and peers [35]. Self-attribution also reinforces this bias [36]. Although overconfidence can lead to excessive trading and price volatility [37,38], AMH suggests that investors may eventually adapt their behavior in response to evolving market dynamics [39].

### Adaptive market hypothesis and adaptability factors

The Adaptive Market Hypothesis (AMH) extends the Efficient Market Hypothesis, reconciling efficient market principles with behavioral biases. It suggests that market efficiency and inefficiency can coexist with intelligent, forward-looking, competitive, and imperfect investors continuously learning from their mistakes and adapting to changing economic conditions [7–9]. In a time-varying efficient market, where market efficiency fluctuates, behavioral anomalies persist but evolve over time [12]. Failure to adapt can result in financial losses and market exit, emphasizing that adaptability is crucial for survival in a competitive market. AMH suggests that retail investors’ behavior, shaped by their adaptation, is determined by both internal and external factors. This study introduces the concept of ‘adaptability factors,’ encompassing *cognitive* (internal) and *social* (external) factors, which represent the mechanisms through which AMH operate at the individual level. Internal adaptability factors include trading experience (EXP), self-reflection (RFL), desire for learning (DL), and financial literacy (FL), while external factors comprise social influence (SCL), consultation with financial advisors (ADV), and media exposure (MED). The adaptability factors proposed in this study are conceptually embedded within the Adaptive Market Hypothesis and bounded rationality framework. AMH emphasizes that investors adapt through learning, feedback, and interaction within competitive and uncertain environments. Internal factors such as trading experience, self-reflection, desire for learning, and financial literacy represent cognitive mechanisms through which investors process feedback and adjust behavior over time. External factors including social influence, consultation with financial advisors, and media exposure reflect environmental information channels that shape adaptive responses under conditions of uncertainty and information asymmetry. Thus, the adaptability factors operationalize AMH at the individual investor level without departing from its evolutionary learning perspective.

Although adaptability is often assumed to support rational adjustments, evidence shows that the same factors may heighten susceptibility to biases. Trading experience and a strong learning desire may fuel overconfidence [40–42], while social influence amplifies HRD [29,43]. Thus, adaptability interacts with cognitive and environmental constraints in line with Bounded Rationality [11]. By modeling adaptability factors as antecedents of HRD and OVR, this study extends AMH beyond its general conceptual framing and provides an empirically testable pathway linking adaptation to specific biases in frontier markets.

#### Trading experience.

Trading experience, typically measured in number of trading years in the stock market, allows investors to accumulate knowledge rapidly [35,44]. Under AMH, experience, gained through reinforcement and reflection, should reduce biases as investors adapt to competitive market conditions [7,8,45]. However, empirical findings are mixed; experience has been found to reduce HRD [26], but also to increase OVR through frequent trading and perceived expertise [37,46,47]. These mixed results justify testing the following hypotheses:

***H1A-B. Trading experience (a) negatively influences herd bias, but (b) positively influences overconfidence bias.***

#### Self-reflection.

Reflection transforms knowledge into learning by integrating emotional and cognitive processes [48]. High self-reflection allows investors to reevaluate and adjust beliefs based on market information, potentially reducing HRD [26,49]. However, excessive confidence in one’s reflective judgments may foster OVR and risk preference [50,42]. This leads to the following hypotheses:

***H2A-B. Self-reflection (a) negatively influences herd bias, but (b) positively influences overconfidence bias.***

#### Desire for learning.

An investor’s learning reflects intrinsic motivation driven by emotional engagements, such as attention, interest, or curiosity [51]. DL can strengthen cognitive processing, leading to improved decision-making and reduced HRD, through awareness of personal limitations [26,52]. However, a strong desire to learn may also elevate OVR if investors overrate their improved reflective capacity [40]. Thus, DL may reduce HRD while simultaneously increasing OVR, leading to the following hypotheses:

***H3A-B. The desire for learning (a) negatively influences herd bias, but (b) positively influences overconfidence bias.***

#### Financial literacy.

Financial literacy, encompassing both basic numerical skills and advanced knowledge of financial concepts, enables individuals to make more rational investment decisions [53–56]. Higher FL can mitigate HRD [57] and reduce OVR by replacing heuristic-based judgments with informed reasoning. Within the AMH framework, this study examines the impact of FL on investors’ behavioral biases, leading to the following hypotheses:

***H4A-B. Financial literacy negatively influences (a) herd bias and (b) overconfidence bias.***

### External adaptability factors and behavioral biases

#### Social influence.

Social influence, including word-of-mouth communication with family, friends, and peer networks, plays a critical role in shaping investment behavior [58,59]. Although such interactions can provide valuable insights, they may also disseminate unreliable or biased information, thereby increasing HRD [29] and occasionally OVR [35]. Accordingly, the following hypotheses are proposed:

***H5A-B. Social influence positively influences (a) herd bias and (b) overconfidence bias.***

#### Consultation with financial advisors.

Financial advisors offer expertise and reduce information search costs, thereby facilitating rational investment [60,61]. Investors often rely on their advice when managing portfolios and making strategic decisions [35,62]. However, advisors’ prioritization of earning brokerage fees may prompt excessive trading and OVR [63]. Thus, ADV may reinforce both HRD and OVR. This leads to the following hypotheses:

***H6A-B. Consultation with financial advisors positively influences (a) herd bias and (b) overconfidence bias.***

#### Media.

Media platforms, including newspapers, television, social media, blogs, and financial articles, are vital sources of information, shaping investment behavior [64,65]. While media can help investors make informed decisions and adapt more efficiently [8,66], excessive media exposure to sensational coverage can amplify HRD and OVR, impacting market returns and volatility [67,68]. Thus, the hypotheses are:

***A-B. Information from media positively influences (a) herd bias and (b) overconfidence bias.***

### Theoretical framework

**Fig 1:**
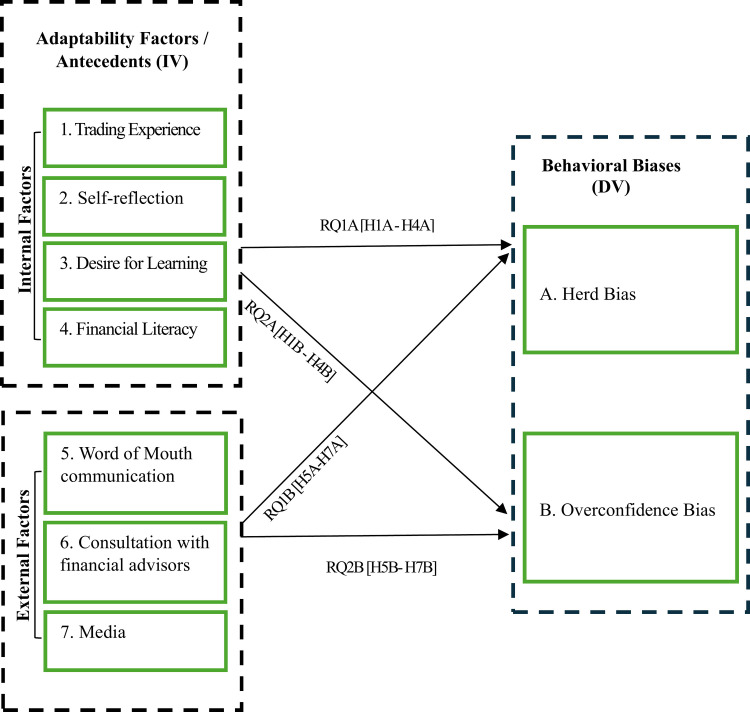
Market adaptability model of individual investors.

This study proposes a Market Adaptability Model (Fig 1) to examine how internal and external adaptability factors influence investors’ behavioral biases, particularly HRD and OVR*.* Behavioral biases, HRD and OVR, serve as dependent variables, while internal (EXP, RFL, DL, FL) and external (SCL, ADV, MED) adaptability factors act as *independent* variables. Grounded in the Adaptive Market Hypothesis (AMH) and Bounded Rationality, the framework suggests that adaptability processes help investors survive in dynamic markets. However, these processes can also influence psychological biases when shaped by cognitive and social factors.

## Materials and methods

### Research design, data collection, and sampling

This study employed an empirical, quantitative research design based on behavioral finance theories. The target population consisted of individual investors trading on the frontier market of the Dhaka Stock Exchange (DSE). The survey was administered using a dual-mode approach, both online and through face-to-face distribution of hardcopy questionnaires. No qualitative interviews were conducted; the term “face-to-face” refers exclusively to the in-person administration of the structured questionnaire. The in-person surveys were conducted during trading hours in collaboration with 11 brokerage houses in Dhaka, where researchers visited brokerage house trading floors and requested eligible investors to complete the questionnaire on-site. The online survey was distributed through Google Forms to investors identified via brokerage houses, who were contacted and invited to participate after receiving study information. Participation was voluntary, with no incentives provided. A screening question confirmed eligibility by verifying a minimum of two years of trading experience. All data analyzed in this research were obtained solely from structured survey responses.

Respondents were selected using non-probability sampling, particularly purposive (judgmental) sampling to ensure representation of active investors. A probability-based sampling frame was not available, as no comprehensive contact list of active investors exists, and probability-based approaches are often impractical in social science research. Access to participants further required collaboration with brokerage houses. Purposive sampling ensured that all respondents met strict eligibility criteria: being at least 18 years old and having two or more years of trading experience, consistent with prior behavioral finance studies [29,69]. To mitigate potential bias, the gender distribution of the sample was aligned with Central Depository Bangladesh Limited (CDBL) records, reflecting a male-to-female ratio of approximately 75:25.

Sample size estimation followed established approaches. In particular, the “10-times rule” [70], based on the maximum number of arrows pointing to a construct in the model (nine), yielded a minimum sample size of 90. Using G*Power [71], with desired statistical power (1–β = 0.80), significance level (α = 0.05), medium effect size (f^2^ = 0.15), and number of predictors = 9, the minimum required sample size was 114. The final dataset of 640 valid responses exceeded both thresholds, strengthening the reliability of results. Data were collected cross-sectionally over a four-month period (15 November 2022–15 February 2023).

### Scale development and validation of questionnaire

Adaptability factors were conceptualized as internal (trading experience, self-reflection, desire for learning, and financial literacy) and external (social influence, consultation with financial advisors, and media) constructs, consistent with the AMH. Unlike prior behavioral finance research that mostly examined isolated biases, this study considers adaptability factors as antecedents shaping herding (HRD) and overconfidence (OVR). In doing so, the study extends AMH beyond its qualitative framing and aligns it with the theory of Bounded Rationality. The structured questionnaire comprised items measuring seven independent variables, two dependent variables, and seven demographic variables. Independent variables were measured as follows: EXP by years of trading [35]; RFL using the 7-item *reflective thinking* scale [51]; DL using the 8-item Self-Directed Learning Readiness Scale [72]; FL using five items [53,73]; SCL with six items [29]; ADV with five items [26]; and MED with a four-item scale derived from various literature [27,29,39]. Dependent variables were HRD, measured with four items [33] and OVR assessed with six items [27]. Demographic variables comprised gender, marital status, age, education level, occupation, monthly income, and region. The marker variable, “attitude toward the color blue (BLU),” a four-item scale, was used to assess common method bias [74]. Most constructs used 5-point Likert scales, except FL (multiple-choice) and EXP (single-item).

Validity and reliability of the survey instrument were established through a two-stage pre-test. First, expert validation was conducted by five experts, and the Content Validity Index (CVI) confirmed both item- and scale-level validity. Next, the pilot survey, conducted with 100 participants, confirmed satisfactory reliability and validity. All constructs exceeded the 0.7 threshold for Cronbach’s alpha (α) and Composite Reliability (CR), and factor loadings were above 0.50 for all items, confirming internal consistency, reliability, and convergent validity [70,75]. Respondents from the pilot test were excluded from the final survey.

### Ethical considerations

Ethical approval was obtained from the Universiti Malaya Research Ethics Committee (UMREC) (Ref: UM.TNC 2/UMREC, 20 January 2022). All participants provided written informed consent prior to participation. They were given detailed information regarding the study objectives, procedures, voluntary participation, and data confidentiality. Consent to publish their data was assumed upon completion of the questionnaire. As the study involved individual investors rather than a formal community or institution, consent from a community representative was not required. The study complies with all relevant ethical regulations, and there were no deviations from the approved study protocol after obtaining ethical clearance from UMREC.

### Data analysis techniques

Data were entered, cleaned, and screened using IBM SPSS Statistics for Windows, version 26.0 [76], retaining all 640 responses. Descriptive statistics and checks of multiple regression assumptions indicated that the data were non-normally distributed. Consequently, Partial Least Squares Structural Equation Modeling (PLS-SEM) was employed in SmartPLS 4.0 [77] to assess relationships among constructs and evaluate the model’s predictive accuracy. PLS-SEM was appropriate given the non-normal data, model complexity, and sample size. The analysis followed a four-step process: (i) testing for normality, (ii) assessing common method variance (CMV), (iii) validating the measurement model, and (iv) evaluating the structural model [70,78].

### Structural Model Evaluation and Statistical Assumptions

Prior to estimating the structural model, key statistical assumptions were assessed to ensure the robustness and validity of the PLS-SEM results. Multivariate normality was tested using Mardia’s skewness and kurtosis, which confirmed non-normality and further justified the use of PLS-SEM. Multicollinearity was evaluated through variance inflation factors (VIF), indicating no collinearity concerns. Linearity and homoscedasticity were verified by residual scatterplots, while Durbin-Watson statistics confirmed the independence of errors. Sample size adequacy was ensured with the final sample of 640 far exceeding minimum requirements. Structural model evaluation was then conducted using non-parametric bootstrapping with 10,000 resamples to generate stable standard errors and 95% bias-corrected confidence intervals. For model evaluation, both explanatory power and predictive adequacy were assessed, where R^2^ values indicated moderate explanatory power, and Q^2^ statistics confirmed moderate predictive relevance of the endogenous constructs. Model fit was evaluated using standardized root mean square residual (SRMR < 0.08), supplemented by additional PLS-specific diagnostics (d_ULS and d_G) used for comparative purposes. These diagnostics supported the robustness, reliability, and theoretical adequacy of the model despite reliance on non-probability sampling and non-normal data distributions. Extended robustness checks are reported in the Results section and in Supporting Information (**S3 Text**, **S4 Table**).

## Results

### Sample characteristics

Socio-demographic characteristics of the individual investors, presented as a frequency distribution, are summarized in **Table 1**. Among the respondents, 75% were male, reflecting the male-to-female investor ratio (75:25) in the DSE. Most respondents were aged between 25–44 years, and over half held a master’s degree. The majority were employed in the private sector and resided in the Dhaka division, including the capital.

**Table 1:** Socio-demographic profile of the investors.

Variable	Category	Frequency	Percentage (%)
Gender	Male	480	75.0
	Female	160	25.0
Marital Status	Married	483	75.5
	Unmarried	152	23.8
	Divorced	5	0.8
Age (in years)	Less than 25 years	46	7.2
	25-34 years	203	31.7
	35-44 years	244	38.1
	45-54 years	99	15.5
	55–64 years	35	5.5
	65 years and above	13	2.0
Education level	High School/SSC/OL	12	1.9
	Senior High School/HSC/AL	25	3.9
	Bachelor’s degree	194	30.3
	Master’s degree	351	54.8
	Professional Course	53	8.3
	Doctorate	5	0.8
Occupation	Private sector employee	422	65.9
	Public sector employee	54	8.4
	Retired	35	5.5
	Self-employed	95	14.8
	Unemployed	34	5.3
Monthly Income	Up to BDT 50,000	264	41.3
	BDT 50,001−1,00,000	195	30.5
	BDT 1,00,001−1,50,000	76	11.9
	1,50,001 - 2,00,000	47	7.3
	2,00,001 and above	58	9.1
State (Region)	Barishal	43	6.7
	Chattogram	117	18.3
	Dhaka	357	55.8
	Khulna	39	6.1
	Mymensingh	20	3.1
	Rajshahi	31	4.8
	Rangpur	20	3.1
	Sylhet	13	2.0

All amounts are reported in Bangladeshi Taka (BDT).

**Source(s):** Authors’ Own Creation.

### Normality testing

A normality test was conducted using Mardia’s multivariate skewness and kurtosis test via Webpower software. The data were not multivariate normal, as both skewness and kurtosis exceeded recommended cut-off values [79]. This justified the use of PLS-SEM, which is robust to non-normal data distributions.

### Common method variance

This study was susceptible to common method variance, given the reliance on a single source of respondents (individual investors) and a single data collection method (questionnaire survey). To address potential CMV, both procedural and statistical remedies were applied. Procedurally, item wording was varied and respondent anonymity was assured to minimize evaluation apprehension. Statistically, “*attitude towards color blue” (BLU)* was used as a marker variable [74]. The Confirmatory Factor Analysis (CFA) Marker Technique showed no significant changes in R^2^ and β values between the method factor model and the baseline models, suggesting the absence of CMV. Additionally, Harman’s Single-Factor Test indicated that percentage variance explained by a single factor was less than 50%, further confirming that CMV was not a concern.

### Measurement model assessment

*Convergent validity*, which assesses the validity of individual items, was evaluated using item loadings, Cronbach’s Alpha, Composite Reliability, and Average Variance Extracted (AVE). Items with loadings below 0.50 were removed prior to final estimation. The measurement model results for convergent and discriminant validity are presented in **Table 2**. All retained item loadings exceeded 0.50, with most above the preferred threshold of 0.708, except for HRD2. Cronbach’s Alpha values were above 0.70 for all constructs (except HRD), while CR values consistently exceeded 0.70. AVE scores for all constructs were greater than 0.50, thus, confirming internal consistency, reliability, and convergent validity.

**Table 2:** Measurement model results for convergent validity and discriminant validity.

Convergent Validity				Discriminant Validity
Constructs	Cronbach’s Alpha	CR	AVE	(HTMT < 0.85)
ADV	0.866	0.904	0.654	Yes
DL	0.789	0.855	0.543	Yes
FL	N/A	N/A	N/A	Yes
EXP	N/A	N/A	N/A	Yes
HRD	0.584	0.766	0.541	Yes
MED	0.732	0.827	0.552	Yes
OVR	0.821	0.87	0.527	Yes
RFL	0.721	0.823	0.541	Yes
SCL	0.702	0.816	0.527	Yes

**Source:** Authors’ Own Creation

**Fig 2:**
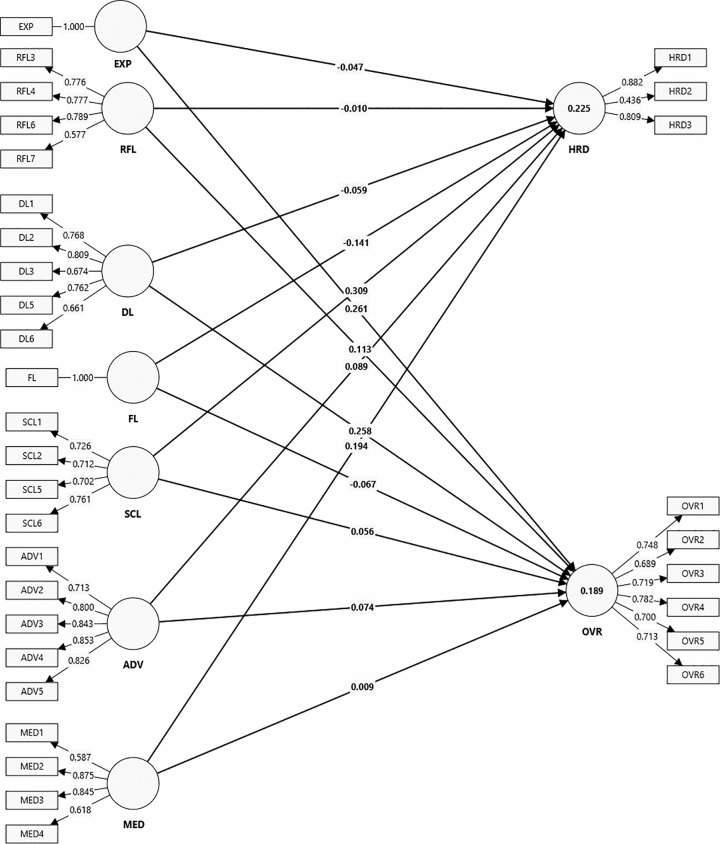
Market adaptability model relating adaptability factors and behavioral biases.

*Discriminant validity*, reflecting the degree to which items are divergent among constructs, was assessed using the Heterotrait-Monotrait (HTMT) ratio [80]. As reported in **Table 2**, all HTMT values were below the 0.85 threshold, which indicates strong discriminant validity and confirms that respondents clearly distinguished among the constructs. Fig 2 illustrates the final market adaptability model linking adaptability factors and behavioral biases.

Collinearity Statistics, measured using the Variance Inflation Factor (VIF) in the inner model, indicate collinearity between independent variables and each endogenous variable. VIF values for the endogenous variables (HRD and OVR) were below 5.0, indicating no multicollinearity among the independent variables [79].

### Structural model testing

The structural model assessed how internal and external adaptability factors influence HRD and OVR. To evaluate the hypothesized relationships, a 10,000-sample re-sample bootstrapping method was applied with a 0.05 significance level using one-tailed testing (critical value = 1.645) to address the non-normality of data [81]. Both p-values and 95% bias-corrected confidence intervals (CI) were reported to ensure robust statistical decisions. The hypotheses test results, representing the direct effects of adaptability factors on herding and overconfidence, are presented in **Table 3**.

**Table 3:** Hypothesis testing of adaptability factors predicting herding and overconfidence biases.

Hypothesis	Path	Std. Beta (β)	p-value	95% BC Confidence Interval	Decision
Herding Bias					
H1A	EXP → HRD	−0.047	0.114	−0.110, 0.016	Not Supported
H2A	RFL → HRD	−0.010	0.423	−0.095, 0.076	Not Supported
H3A	DL → HRD	−0.059	0.085	−0.129, 0.012	Not Supported
H4A	FL → HRD	−0.141	<0.001	−0.197, −0.084	**Supported**
H5A	SCL → HRD	0.309	<0.001	0.240, 0.368	**Supported**
H6A	ADV → HRD	0.089	0.010	0.022, 0.150	**Supported**
H7A	MED → HRD	0.194	<0.001	0.124, 0.260	**Supported**
Overconfidence Bias					
H1B	EXP → OVR	0.261	<0.001	0.192, 0.328	**Supported**
H2B	RFL → OVR	0.113	0.024	0.015, 0.203	**Supported**
H3B	DL → OVR	0.258	<0.001	0.181, 0.326	**Supported**
H4B	FL → OVR	−0.067	0.038	−0.126, −0.003	**Supported**
H5B	SCL → OVR	0.056	0.103	−0.015, 0.131	Not Supported
H6B	ADV → OVR	0.074	0.067	−0.009, 0.154	Not Supported
H7B	MED → OVR	0.009	0.423	−0.069, 0.084	Not Supported

**Source(s):** Authors’ Own Creation.

**Notes:**

■ BC Confidence Interval = Bias-Corrected 95% Confidence Interval.

■ Bolded hypotheses indicate statistically supported relationships (p < 0.05, one-tailed).

The results reveal significant relationships between adaptability factors and behavioral biases in stock market investments. All external adaptability factors, SCL, ADV, and MED, positively influenced herding (*β* = 0.309, p < 0.001; *β* = 0.089, p < 0.05; *β* = 0.194, p < 0.001 respectively). Internal adaptability factors, EXP, RFL, and DL, had significant positive effects on overconfidence (*β* = 0.261, p < 0.001; *β* = 0.113, p = 0.024; and *β* = 0.258, p < 0.001 respectively). By contrast, FL had a significant negative effect on both herding (*β* = −0.141, p < 0.001) and overconfidence (*β* = −0.067, p < 0.05). All supported hypotheses had confidence intervals excluding zero at the 5% significance level, confirming their statistical significance. The output patterns suggest that adaptability factors operate through distinct pathways: social influences primarily promote imitation (herding), whereas cognitive processes enhance self-assessment while also increasing overconfidence.

### Predictive relevance

The predictive accuracy of the model was assessed through the coefficient of determination score (R^2^). Both herding and overconfidence demonstrated ***moderate*** accuracy with *R*^*2*^ values of 0.225 and 0.189 respectively [70], indicating that the adaptability factors explain meaningful variation in these biases.

### Robustness and model diagnostics

To ensure the robustness of the structural model, multiple diagnostic checks were conducted within the PLS-SEM framework (**Table 4**). Non-parametric bootstrapping with 10,000 subsamples confirmed the stability of all significant paths, with 95% bias-corrected confidence intervals excluding zero, thereby ensuring the reliability of parameter estimates. Model fit was acceptable (SRMR = 0.072 for the saturated model; 0.073 for the estimated model), with moderate explanatory power (R^2^ = 0.225 for herding; 0.189 for overconfidence) and predictive relevance (Q^2^ > 0 for both endogenous constructs). Collinearity checks confirmed that all VIF values were below 5, ruling out multicollinearity. Linearity and homoscedasticity were supported by residual scatterplots, and Durbin-Watson statistics indicated independence of errors. Additional PLS-SEM fit diagnostics, including d_ULS (2.946; 3.007) and d_G (0.606; 0.608), further supported the adequacy of the model. Extended diagnostics are provided in **S3 Text** and **S4 Table**. Collectively, these diagnostics indicate that the model was robust, statistically reliable, and theoretically meaningful, even under purposive (non-probability) sampling and non-normal data distributions.

**Table 4:** PLS-SEM model fit and predictive diagnostics.

Index	Saturated Model	Estimated Model	Notes/ Thresholds
SRMR	0.072	0.073	<0.08 acceptable
d_ULS	2.946	3.007	Comparative only
d_G	0.606	0.608	Comparative only
R² (Herding)	0.225	–	Moderate explanatory power
R² (Overconfidence)	0.189	–	Moderate explanatory power
Q² (Herding)	> 0	–	Predictive relevance confirmed
Q² (Overconfidence)	> 0	–	Predictive relevance confirmed
VIF (all constructs)	< 5	–	No multicollinearity
Durbin-Watson	~2.0	–	No autocorrelation of errors
Residual plots	–	–	Linearity, homoscedasticity ok

**Source:** Authors’ Own Creation.

## Discussion

This study examined how adaptability factors shape behavioral biases among individual investors in the frontier stock market of Bangladesh. The results reveal a dual pathway of bias formation: external adaptability factors such as social influence, consultation with financial advisors, and media strongly drive herding, while internal factors including trading experience, self-reflection, and desire for learning primarily fuel overconfidence. Financial literacy, an internal factor, notably reduces both herding and overconfidence, underscoring its role in fostering more rational decision-making. These findings directly address the study’s research questions by confirming that external adaptability factors predominantly influence herding (RO1), while internal adaptability factors shape overconfidence (RO2), with financial literacy mitigating both biases and thereby extending the application of AMH to frontier markets.

Interpreted through the Adaptive Market Hypothesis (AMH), these findings show that investors adapt heuristically to survive under uncertain market conditions [8,12]. When informational frictions are high, external cues dominate, leading investors to imitate others as a short-run survival strategy. This aligns with prior empirical evidence documenting a positive relationship between social influence and herding behavior across frontier markets [21,25,26,82–85]. In the specific context of Bangladesh, Khan and Tan (2020) [83] demonstrate that strong family and social ties significantly intensify herd behavior among retail investors, reinforcing the context-sensitive nature of adaptive imitation. Similarly, reliance on financial advisors has been shown to amplify collective trading tendencies, thereby intensifying herding dynamics in frontier markets [86]. Moreover, media-driven sentiment intensifies herd reactions in volatile markets by spreading sensationalized or incomplete information, as documented in studies examining information diffusion and investor reactions [43,68,87,88].

Internal learning processes, such as trading experience or self-reflection, enhance adaptive capacity but often result in overconfidence when investors overestimate their competence [40,89,90]. While experiential learning and self-reflection may support investment success [91,92], extensive evidence indicates that the assumption that frequent trading or accumulated experience equates to superior skill has repeatedly been shown to inflate overconfidence [26,93]. Empirical studies consistently report a positive association between trading experience and overconfidence [34]. Similarly, cognitive reflection research shows that increased self-perceived competence may lead to miscalibrated self-assessment and amplify overconfidence [40,52]. In contrast, financial literacy reduces reliance on heuristic shortcuts, lowering susceptibility to both herding and overconfidence. Empirical evidence consistently demonstrates that higher financial literacy lowers herd tendencies and mitigates overconfidence bias [18,20,27].

Regarding nonsignificant hypotheses, internal adaptability factors other than financial literacy were not significantly related to herding, and external factors were not significantly associated with overconfidence. Prior research suggests that experience and learning can produce mixed behavioral effects, either reducing imitation or strengthening reliance on personal judgment [40,94]. Evidence that experienced investors prefer autonomous decision-making over advisory dependence [33,95**]** further supports the absence of significant associations.

These findings reinforce AMH’s principle that adaptation is context-specific and shaped by feedback loops in volatile environments [8]. In the Bangladeshi context, where regulatory oversight is weak and information asymmetry remains pervasive, adaptability produces mixed outcomes: it aids survival but simultaneously reinforces biases. By empirically testing how adaptability factors interact with herding and overconfidence, this study demonstrates that bounded adaptability, rather than pure rationality, constrains investor decision-making in frontier markets [96,97]. Therefore, the central premise of the Adaptive Market Hypothesis—that markets evolve similarly to biological systems—is especially evident in frontier markets, where investor adaptation supports survival but does not guarantee rational decision-making [7].

### Theoretical Implications

This study extends behavioral finance by integrating internal and external adaptability factors into AMH. While AMH emphasizes investor learning and survival in changing environments [8], its application to specific behavioral biases has been limited [98,99]. Findings show that adaptability is not uniformly beneficial: external adaptability supports short-term survival through imitation, while internal adaptability promotes long-term learning but can also lead to overconfidence in some cases, except for financial literacy, which mitigates both biases. This duality refines AMH’s learning–survival framework, signifying that adaptation does not necessarily yield rationality, particularly in frontier markets with limited institutional support [12].

This study importantly integrates the Adaptive Market Hypothesis and Bounded Rationality into a unified explanatory framework. While AMH explains how investors adjust their behavior through learning and environmental feedback, Bounded Rationality clarifies why such adaptive processes remain constrained by limited cognitive capacity and imperfect information. By empirically modeling herd and overconfidence biases as behavioral outcomes of internal and external adaptability factors, this study demonstrates that adaptive learning operates within bounded cognitive limits. Thus, adaptation and bounded rationality jointly shape investor behavior, particularly in frontier markets characterized by uncertainty and information asymmetry.

Building on this integrated framework, **Table 5** compares adaptability–bias dynamics across frontier, emerging, and developed markets. In the frontier market of Bangladesh, weak oversight and volatility amplify external herding drivers, while internal learning processes increase overconfidence. Financial literacy reduces both herding and overconfidence, confirming its protective role. These results align with prior studies linking social and media influence to herding [43,100] and trading experience or reflection to overconfidence [41,93]. The mitigating effect of literacy echoes findings from Pakistan [101], and emerging markets such as India and Saudi Arabia [13,15], where it reduces overconfidence. In developed markets, greater institutional transparency reduces herding [102,103], while overconfidence is situational, often surfacing during crises [104]. Overall, these findings address gaps highlighted in the literature (Section 2), particularly the underexplored role of adaptability factors in bias formation under AMH, the dual nature of adaptation, and the contextual distinctiveness of frontier markets.

**Table 5:** Comparative dynamics of adaptability factors and behavioral biases across frontier, emerging, and developed markets.

Market Type	Key Adaptability Drivers	Impact on Herding	Impact on Overconfidence
Frontier (Bangladesh, Sri Lanka, Pakistan)	External (SCL, ADV, MED); Internal (EXP, RFL, DL)	Strongly driven by external factors	Internal factors amplify OVR
Emerging (Kenya, India, Saudi Arabia)	Internal dominant (EXP, RFL); External less impactful	Mixed; HRD less externally driven	Internal factors raise OVR; FL moderates
Developed (Spain, Portugal, G7 countries)	High systemic transparency	Low HRD due to regulatory clarity	Low OVR via low trade volume and return dynamics

**Source(s):** Authors’ Own Compilation.

Overall, these implications show that AMH remains a valuable lens across market contexts but must be operationalized with attention to institutional quality and investor literacy. Modeling adaptability factors as distinct cognitive and social mechanisms clarifies why the same learning processes that support survival can also reinforce behavioral biases in volatile, low-transparency environments. This contextualized extension of AMH highlights the need for theoretical and empirical work that explicitly examines how institutional and informational conditions shape the overall impact of adaptability on rationality. Collectively, the study addresses gaps highlighted in the literature (Section 2), particularly the underexplored role of adaptability factors in bias formation under AMH, the dual nature of adaptation, and the contextual distinctiveness of frontier markets.

### Managerial implications

The findings provide several actionable insights for regulators, market institutions, brokerage firms, and listed companies in Bangladesh’s frontier stock market. *First,* the Bangladesh Securities and Exchange Commission (BSEC) should strengthen market oversight, transparency, and disclosure practices to reduce reliance on informal cues and rumor-based trading. In collaboration with the Bangladesh Institute of Capital Market (BICM), financial literacy initiatives should explicitly address behavioral biases and incorporate lessons from past market crises (e.g., 1996 and 2010 crashes) to highlight common pitfalls. *Second,* brokerage firms can establish localized *Investor Literacy Corners* and organize scenario-based workshops or simulated trading sessions. Regular “*Bias Literacy Programs*,” offered in Bangla, would enable investors to recognize their own cognitive biases in realistic decision-making contexts. *Third,* gender-focused initiatives are essential. Tailored literacy and digital programs, delivered through grassroots institutions and designed for female investors, can address psychological barriers, improve confidence, and expand women’s participation in capital markets. *Fourth,* technology-driven nudges via mobile applications can incorporate behavioral alerts, such as warnings about herd-driven price swings or excessive trading, to help investors avoid impulsive decisions. *Finally,* listed companies should ensure timely and accurate dissemination of financial information to limit speculation and herd-driven volatility, thereby promoting more stable market behavior.

Taken together, these measures address the dual role of adaptability identified in this study. While external adaptability encourages herding and internal adaptability (excluding financial literacy) fuels overconfidence, targeted regulatory, educational, and technological interventions can alleviate these biases and build a more resilient, inclusive, and investor-friendly frontier market.

## Conclusion

Over recent decades, individual investors’ behavioral rationality has been challenged due to reliance on cognitive heuristics. This study investigated the impact of adaptability factors on behavioral biases, within Bangladesh’s frontier stock market. In light of AMH and bounded rationality, it fills a key gap in the literature by empirically testing how adaptability factors shape behavioral biases in a dynamic, yet volatile and low-transparency environment. To the best of the researcher’s knowledge, this is the first empirical study to categorize individual investors’ adaptability into internal and external factors and examine their distinct effects on herding and overconfidence in a frontier market.

Findings reveal that external adaptability factors primarily influence herding and internal factors drive overconfidence. Financial literacy is the only factor that consistently reduces both behavioral biases. These insights suggest that improving adaptability, particularly through structured financial knowledge, can help individual investors minimize biased decision-making. The study highlights the need for targeted financial education and policy measures tailored to frontier market conditions, where behavioral biases are amplified by institutional weaknesses and information asymmetry. Overall, this study contributes to behavioral finance and AMH literature by demonstrating how adaptability factors shape biases in frontier markets, providing both theoretical and practical pathways for building a more resilient financial system.

### Limitations

This study has several limitations that should be considered when interpreting the findings. *First,* obtaining authentic responses from Bangladeshi female investors posed challenges due to cultural and market practices, although careful screening was applied to ensure genuine participants. *Second,* reliance on self-reported perceptual data may have been influenced by respondent mood or context, introducing potential subjectivity, despite diagnostic checks confirming the nonexistence of major bias. *Third,* the study focused only on individual investors, excluding institutional participants whose adaptability and behavioral patterns may differ. Fourth, the study employed purposive rather than probability sampling, due to the absence of a comprehensive sampling frame of active retail traders. While this limits generalizability, potential bias was mitigated by aligning the sample’s gender distribution with CDBL statistics and by conducting extensive PLS-SEM diagnostics-including bootstrap resampling, multicollinearity checks, error independence tests, and model fit indices)-which confirmed the stability and validity of the structural relationships. Finally, since the study is cross-sectional, causal relationships between adaptability factors and behavioral biases cannot be definitively established.

### Further research

The study offers several recommendations for future research. *First,* large-scale research with more diverse and representative respondents would enhance validity and generalizability of the findings. *Second,* future studies should explore the relative strength of individual adaptability factors on herding and overconfidence biases, identifying which elements exert the most significant influence. *Third,* studies incorporating moderating and mediating variables would provide deeper insights into how market adaptability influences heuristics. *Fourth*, comparative studies across frontier and emerging markets would clarify the role of cultural and institutional contexts in shaping investor behavior. Finally, longitudinal or experimental designs are recommended to establish stronger causal inferences between adaptability factors, behavioral biases, and irrational investment decisions**.**

## Supporting information

S1 Data Anonymized dataset of 640 individual investors’ survey responses. (XLSX)

S1 Table Socio-demographic profile of the investors. (DOCX)

S2 Table Measurement model results for convergent validity and discriminant validity. (DOCX)

S3 Table Hypothesis Testing of Adaptability Factors Predicting Herding and Overconfidence Biases. (DOCX)

S3 Text Extended Robustness and Model Diagnostics. Non-parametric bootstrapping (10,000 resamples) was applied to generate stable standard errors and 95% bias-corrected confidence intervals for all structural paths. Model fit indices included SRMR (saturated = 0.072; estimated = 0.073), d_ULS (2.946; 3.007), and d_G (0.606; 0.608), supporting overall adequacy. The model showed moderate explanatory power (R^2^ = 0.225 for herding; 0.189 for overconfidence) and positive Q^2^ values, indicating predictive relevance. VIF (< 5) confirmed absence of multicollinearity, while residual diagnostics supported linearity and homoscedasticity. Durbin-Watson statistics further indicated no autocorrelation of errors. These extended diagnostics reinforce that the model was robust, well specified, and theoretically meaningful despite reliance on purposive sampling.
(DOCX)

S4 Table PLS-SEM model fit and predictive diagnostics. (DOCX)

S5 Table Comparative dynamics of adaptability factors and behavioral biases across frontier, emerging, and developed markets. (DOCX)

S1 Fig Market adaptability model of individual investors. (TIF)

S2 Fig Market adaptability model relating adaptability factors and behavioral biases. (TIF)

S1 File Questionnaire. Completed PLOS inclusivity in global research questionnaire.
(DOCX)

## References

[1] Fama EF. Portfolio analysis in a stable Paretian market. Manage Sci. 1965;11(3):404–19. doi: 10.1287/mnsc.11.3.404

[2] Markowitz H. The utility of wealth. J Polit Econ. 1952;60(2):151–8. doi: 10.1086/257177

[3] Kubilay B, Bayrakdaroglu A. An empirical research on investor biases in financial decision-making, financial risk tolerance and financial personality. Int J Financ Res. 2016;7(2):171–82. doi: 10.5430/ijfr.v7n2p171

[4] De Bortoli D, da Costa N Jr, Goulart M, Campara J. Personality traits and investor profile analysis: A behavioral finance study. PLoS One. 2019;14(3):e0214062. doi: 10.1371/journal.pone.0214062 30917175 PMC6436746

[5] Kalahasthi R, Bhuptani PH, Kapoor H. An analysis of thoughts, behaviours, and emotions in daily decision-making. Psychol Stud. 2017;62(4):409–20. doi: 10.1007/s12646-017-0430-x

[6] Shah SZ, Ahmad M, Mahmood F. Heuristic biases in investment decision-making and perceived market efficiency: A survey at the Pakistan stock exchange. Qual Res Financ Mark. 2018;10(1):85–110. doi: 10.1108/QRFM-04-2017-0033

[7] Lo AW. The adaptive markets hypothesis: Market efficiency from an evolutionary perspective. J Portf Manag. 2004;30(5):15–29. doi: 10.3905/jpm.2004.442611

[8] Lo AW. Reconciling efficient markets with behavioral finance: The adaptive markets hypothesis. J Invest Consult. 2005;7(2):21–44.

[9] Lo AW. Adaptive markets and the new world order. Financ Anal J. 2012;68(2):18–29. doi: 10.2469/faj.v68.n2.6

[10] Hull M, McGroarty F. Do emerging markets become more efficient as they develop? Long memory persistence in equity indices. Emerg Mark Rev. 2014;18:45–61. doi: 10.1016/j.ememar.2013.11.001

[11] Simon HA. A behavioral model of rational choice. Q J Econ. 1955;69(1):99–118. doi: 10.2307/1884852

[12] Akhter T, Yong O. Adaptive market hypothesis and momentum effect: Evidence from Dhaka Stock Exchange. Cogent Economics & Finance. 2019;7(1):1650441. doi: 10.1080/23322039.2019.1650441

[13] Frontiers Editorial Office. Retraction: Post-COVID-19 investor psychology and individual investment decision: a moderating role of information availability. Front Psychol. 2025;16:1675381. doi: 10.3389/fpsyg.2025.1675381 40851630 PMC12367790

[14] Rubesam A, Raimundo G de S. Covid-19 and herding in global equity markets. J Behav Exp Finance. 2022;35:100672. doi: 10.1016/j.jbef.2022.100672 35694369 PMC9167689

[15] Seraj AHA, Alzain E, Alshebami AS. The roles of financial literacy and overconfidence in investment decisions in Saudi Arabia. Front Psychol. 2022;13:1005075. doi: 10.3389/fpsyg.2022.1005075 36248580 PMC9565838

[16] Vidya CT, Ravichandran R, Deorukhkar A. Exploring the effect of Covid-19 on herding in Asian financial markets. MethodsX. 2023;10:101961. doi: 10.1016/j.mex.2022.101961 36507467 PMC9721475

[17] Economou F. Herding in frontier markets: evidence from the Balkan region. RBF. 2019;12(2):119–35. doi: 10.1108/rbf-08-2018-0090

[18] Iram T, Bilal AR, Ahmad Z. Investigating The Mediating Role of Financial Literacy on The Relationship Between Women Entrepreneurs’ Behavioral Biases and Investment Decision Making. GADJAH MADA INT J BUS. 2023;25(1):93. doi: 10.22146/gamaijb.65457

[19] MSCI. Market classification framework. MSCI Inc. 2024. https://www.msci.com/our-solutions/indexes/market-classification

[20] Haque ME, Imam MO. Investor Psychology in the Bangladesh Equity Market: An Examination of Herding Behavior Across Diverse Market States. Risks. 2025;13(4):78. doi: 10.3390/risks13040078

[21] Muktadir-Al-Mukit D. Do sociodemographic factors have influence on risk tolerance level of stock market investors? An analysis from a developing country perspective. SAJBS. 2020;11(2):149–73. doi: 10.1108/sajbs-11-2019-0193

[22] Hossain T, Siddiqua P. Exploring the influence of behavioral aspects on stock investment decision-making: a study on Bangladeshi individual investors. PSU Res Rev. 2024;8(2):467–83. doi: 10.1108/PRR-10-2021-0054

[23] Wang W, Guo L, Sun R. Rational herd behavior in online learning: insights from MOOC. Comput Hum Behav. 2019;92:660–9. doi: 10.1016/j.chb.2017.10.009

[24] Yu H, Dan M, Ma Q, Jin J. They all do it, will you? Event-related potential evidence of herding behavior in online peer-to-peer lending. Neurosci Lett. 2018;681:1–5. doi: 10.1016/j.neulet.2018.05.021 29772258

[25] Guney Y, Kallinterakis V, Komba G. Herding in frontier markets: Evidence from African stock exchanges. Journal of International Financial Markets, Institutions and Money. 2017;47:152–75. doi: 10.1016/j.intfin.2016.11.001

[26] Shantha KVA. Individual Investors’ Learning Behavior and Its Impact on Their Herd Bias: An Integrated Analysis in the Context of Stock Trading. Sustainability. 2019;11(5):1448. doi: 10.3390/su11051448

[27] Baker HK, Kumar S, Goyal N, Gaur V. How financial literacy and demographic variables relate to behavioral biases. MF. 2019;45(1):124–46. doi: 10.1108/mf-01-2018-0003

[28] Caglayan M, Talavera O, Zhang W. Herding behaviour in P2P lending markets. Journal of Empirical Finance. 2021;63:27–41. doi: 10.1016/j.jempfin.2021.05.005

[29] Kumari S, Chandra B, Pattanayak JK. Personality traits and motivation of individual investors towards herding behaviour in Indian stock market. K. 2019;49(2):384–405. doi: 10.1108/k-11-2018-0635

[30] Jain J, Walia N, Gupta S. Evaluation of behavioral biases affecting investment decision making of individual equity investors by fuzzy analytic hierarchy process. RBF. 2019;12(3):297–314. doi: 10.1108/rbf-03-2019-0044

[31] Abreu M. How biased is the behavior of the individual investor in warrants?. Res Int Bus Financ. 2019;47:139–49. doi: 10.1016/j.ribaf.2018.07.006

[32] Ahmad M, Shah SZ. Overconfidence heuristic-driven bias in investment decision-making and performance: mediating effects of risk perception and moderating effects of financial literacy. J Econ Adm Sci. 2022;38(1):60–90. doi: 10.1108/JEAS-07-2020-0116

[33] Metawa N, Hassan MK, Metawa S, Safa MF. Impact of behavioral factors on investors’ financial decisions: case of the Egyptian stock market. IMEFM. 2019;12(1):30–55. doi: 10.1108/imefm-12-2017-0333

[34] Kansal P, Singh S. Determinants of overconfidence bias in Indian stock market. Qual Res Financ Mark. 2018;10(4):381–94. doi: 10.1108/QRFM-03-2017-0015

[35] Abreu M, Mendes V. Information, overconfidence and trading: Do the sources of information matter?. Journal of Economic Psychology. 2012;33(4):868–81. doi: 10.1016/j.joep.2012.04.003

[36] Mushinada VNC, Veluri VSS. Investors overconfidence behaviour at Bombay Stock Exchange. IJMF. 2018;14(5):613–32. doi: 10.1108/ijmf-05-2017-0093

[37] Barber BM, Odean T. Trading Is Hazardous to Your Wealth: The Common Stock Investment Performance of Individual Investors. The Journal of Finance. 2000;55(2):773–806. doi: 10.1111/0022-1082.00226

[38] Pikulina E, Renneboog L, Tobler PN. Overconfidence and investment: An experimental approach. Journal of Corporate Finance. 2017;43:175–92. doi: 10.1016/j.jcorpfin.2017.01.002

[39] Mushinada VN. Are individual investors irrational or adaptive to market dynamics?. J Behav Exp Financ. 2020;25:100243. doi: 10.1016/j.jbef.2019.100243

[40] Sanchez C, Dunning D. Overconfidence among beginners: Is a little learning a dangerous thing?. J Pers Soc Psychol. 2018;114(1):10–28. doi: 10.1037/pspa0000102 29094960

[41] Ikram B, Fouad BE, Sara C. An exploration of overconfidence and the disposition effect in the stock market. Int J Financ Stud. 2023;11(2):78. doi: 10.3390/ijfs11020078

[42] Mata A. Overconfidence in the Cognitive Reflection Test: Comparing Confidence Resolution for Reasoning vs. General Knowledge. J Intell. 2023;11(5):81. doi: 10.3390/jintelligence11050081 37233330 PMC10219213

[43] Yoon J, Oh G. Investor herding behavior in social media sentiment. Front Phys. 2022;10. doi: 10.3389/fphy.2022.1023071

[44] Chen G, Kim KA, Nofsinger JR, Rui OM. Trading performance, disposition effect, overconfidence, representativeness bias, and experience of emerging market investors. J Behav Decis Mak. 2007;20(4):425–51. doi: 10.1002/bdm.561

[45] Hoffmann AOI, Post T. How does investor confidence lead to trading? Linking investor return experiences, confidence, and investment beliefs. J Behav Exp Financ. 2016;12:65–78. doi: 10.1016/j.jbef.2016.09.003

[46] Lan Q, Xiong Q, He L, Ma C. Individual investment decision behaviors based on demographic characteristics: Case from China. PLoS One. 2018;13(8):e0201916. doi: 10.1371/journal.pone.0201916 30092101 PMC6085059

[47] Prosad JM, Kapoor S, Sengupta J. Behavioral biases of Indian investors: a survey of Delhi-NCR region. Qual Res Financ Mark. 2015;7(3):230–63. doi: 10.1108/QRFM-04-2014-0012

[48] Dewey J. How we think: A restatement of the relation of reflective thinking to the educative process. 8th ed. Boston: D.C. Heath. 1933.

[49] Corgnet B, DeSantis M, Porter D. Information aggregation and the cognitive make-up of market participants. Eur Econ Rev. 2021;133:103667. doi: 10.1016/j.euroecorev.2021.103667

[50] Mallek RS, Albaity M. Individual differences and cognitive reflection across gender and nationality the case of the United Arab Emirates. Cogent Economics & Finance. 2019;7(1):1567965. doi: 10.1080/23322039.2019.1567965

[51] Shantha KVA, Xiaofang C, Gamini LPS, McMillan D. A conceptual framework on individual investors’ learning behavior in the context of stock trading: An integrated perspective. Cogent Economics & Finance. 2018;6(1). doi: 10.1080/23322039.2018.1544062

[52] Coutinho MVC, Thomas J, Alsuwaidi ASM, Couchman JJ. Dunning-Kruger Effect: Intuitive Errors Predict Overconfidence on the Cognitive Reflection Test. Front Psychol. 2021;12:603225. doi: 10.3389/fpsyg.2021.603225 33897524 PMC8060648

[53] Almenberg J, Dreber A. Gender, stock market participation and financial literacy. Econ Lett. 2015;137:140–2. doi: 10.1016/j.econlet.2015.10.009

[54] Chen C, Ishfaq M, Ashraf F, Sarfaraz A, Wang K. Mediating Role of Optimism Bias and Risk Perception Between Emotional Intelligence and Decision-Making: A Serial Mediation Model. Front Psychol. 2022;13:914649. doi: 10.3389/fpsyg.2022.914649 35719601 PMC9204191

[55] Jain R, Sharma D, Behl A, Tiwari AK. Investor personality as a predictor of investment intention–mediating role of overconfidence bias and financial literacy. Int J Emerg Mark. 2023;18(12):5680–706. doi: 10.1108/IJOEM-12-2021-1885

[56] Lusardi A, Mitchell OS, Curto V. Financial literacy among the young. J Consum Aff. 2010;44(2):358–80. doi: 10.1111/j.1745-6606.2010.01173.x

[57] Jonsson S, Söderberg I-L, Wilhelmsson M. An investigation of the impact of financial literacy, risk attitude, and saving motives on the attenuation of mutual fund investors’ disposition bias. MF. 2017;43(3):282–98. doi: 10.1108/mf-10-2015-0269

[58] Nohl AM. Typical phases of transformative learning: A practice-based model. Adult Educ Q. 2015;65(1):35–49. doi: 10.1177/0741713614558582

[59] Tauni MZ, Rao Z-R, Fang H-X, Gao M. Does investor personality moderate the relationship between information sources and trading behavior? Evidence from Chinese stock market. MF. 2017;43(5):545–66. doi: 10.1108/mf-08-2015-0231

[60] Clark-Murphy M, Soutar GN. Do retail stockbrokers understand clients’ investment preferences?. J Financ Serv Mark. 2008;13(2):135–49. doi: 10.1057/fsm.2008.11

[61] Tauni MZ, Majeed MA, Mirza SS, Yousaf S, Jebran K. Moderating influence of advisor personality on the association between financial advice and investor stock trading behavior. IJBM. 2018;36(5):947–68. doi: 10.1108/ijbm-10-2016-0149

[62] Kramer MM. Financial literacy, confidence and financial advice seeking. J Econ Behav Organ. 2016;131:198–217. doi: 10.1016/j.jebo.2016.08.016

[63] Mullainathan S, Noeth M, Schoar A. The market for financial advice: An audit study. National Bureau of Economic Research. 2012. doi: 10.3386/w17929

[64] Bondia R, Biswal PC, Panda A. The unspoken facets of buying by individual investors in Indian stock market. RBF. 2019;11(3):324–51. doi: 10.1108/rbf-12-2017-0121

[65] Chen M-Y, Chen T-H. Modeling public mood and emotion: Blog and news sentiment and socio-economic phenomena. Future Generation Computer Systems. 2019;96:692–9. doi: 10.1016/j.future.2017.10.028

[66] Haritha PH, Uchil R. Impact of investor sentiment on decision-making in Indian stock market: an empirical analysis. J Adv Manag Res. 2020;17(1):66–83. doi: 10.1108/JAMR-03-2019-0041

[67] Akhtar F, Thyagaraj KS, Das N. The impact of social influence on the relationship between personality traits and perceived investment performance of individual investors. International Journal of Managerial Finance. 2017;14(1):130–48. doi: 10.1108/ijmf-05-2016-0102

[68] Griffith J, Najand M, Shen J. Emotions in the Stock Market. Journal of Behavioral Finance. 2019;21(1):42–56. doi: 10.1080/15427560.2019.1588275

[69] Ul Abdin SZ, Qureshi F, Iqbal J, Sultana S. Overconfidence bias and investment performance: A mediating effect of risk propensity. Borsa Istanbul Review. 2022;22(4):780–93. doi: 10.1016/j.bir.2022.03.001

[70] Hair JF, Hult GTM, Ringle CM, Sarstedt M. A primer on partial least squares structural equation modeling (PLS-SEM). 3rd ed. Sage Publications. 2021. doi: 10.1007/978-3-030-80519-7

[71] Faul F, Erdfelder E, Lang A-G, Buchner A. G*Power 3: a flexible statistical power analysis program for the social, behavioral, and biomedical sciences. Behav Res Methods. 2007;39(2):175–91. doi: 10.3758/bf03193146 17695343

[72] Fisher MJ, King J. The Self-Directed Learning Readiness Scale for nursing education revisited: a confirmatory factor analysis. Nurse Educ Today. 2010;30(1):44–8. doi: 10.1016/j.nedt.2009.05.020 19541394

[73] Lusardi A, Mitchell OS. Baby boomer retirement security: The roles of planning, financial literacy, and housing wealth. J Monet Econ. 2007;54(1):205–24. doi: 10.1016/j.jmoneco.2006.12.001

[74] Miller BK, Simmering MJ. Attitude Toward the Color Blue: An Ideal Marker Variable. Organizational Research Methods. 2022;26(3):409–40. doi: 10.1177/10944281221075361

[75] Fornell C, Larcker DF. Evaluating structural equation models with unobservable variables and measurement error. J Mark Res. 1981;18(1):39–50. doi: 10.1177/002224378101800104

[76] IBM SPSS Statistics for Windows, version 26.0. Armonk, NY: IBM Corp. 2018.

[77] Ringle CM, Wende S, Becker JM. SmartPLS 4. Oststeinbek: SmartPLS GmbH. 2022.

[78] Sarstedt M, Ringle CM, Hair JF. Partial Least Squares Structural Equation Modeling. Handbook of Market Research. Springer International Publishing. 2021. p. 587–632. doi: 10.1007/978-3-319-57413-4_15

[79] Hair JF, Hult GTM, Ringle C, Sarstedt M. A primer on partial least squares structural equation modeling (PLS-SEM). 2nd ed. Thousand Oaks, CA: Sage Publications. 2017.

[80] Franke G, Sarstedt M. Heuristics versus statistics in discriminant validity testing: a comparison of four procedures. INTR. 2019;29(3):430–47. doi: 10.1108/intr-12-2017-0515

[81] Becker JM, Cheah JH, Gholamzade R, Ringle CM, Sarstedt M. PLS-SEM’s most wanted guidance. Int J Contemp Hosp Manag. 2023;35(1):321–46. doi: 10.1108/IJCHM-04-2022-0474

[82] Speidell LS. Investing in the Unknown and the Unknowable—Behavioral Finance in Frontier Markets. Journal of Behavioral Finance. 2009;10(1):1–8. doi: 10.1080/15427560902719323

[83] Khan MT, Tan SH. Does family affect financial outcomes and psychological biases? Evidence from small investors in Bangladesh. J Fam Bus Manag. 2020;10(2):167–86. doi: 10.1108/JFBM-05-2019-0036

[84] Vosylis R, Erentaitė R. Linking family financial socialization with its proximal and distal outcomes: Which socialization dimensions matter most for emerging adults’ financial identity, financial behaviors, and financial anxiety? Emerg Adulthood. 2020;8(6):464–75. doi: 10.1177/2167696819856763

[85] Tauni MZ, Fang HX, Iqbal A. The role of financial advice and word-of-mouth communication on the association between investor personality and stock trading behavior: Evidence from Chinese stock market. Pers Individ Dif. 2017;108:55–65. doi: 10.1016/j.paid.2016.11.048

[86] Tahidur Rahman Md. Stock Market Crash in Bangladesh: The Moneymaking Psychology of Domestic Investors. AJTAB. 2017;3(3):43. doi: 10.11648/j.ajtab.20170303.12

[87] Fang L, Peress J. Media Coverage and the Cross‐section of Stock Returns. The Journal of Finance. 2009;64(5):2023–52. doi: 10.1111/j.1540-6261.2009.01493.x

[88] Tetlock PC. Does Public Financial News Resolve Asymmetric Information?. Rev Financ Stud. 2010;23(9):3520–57. doi: 10.1093/rfs/hhq052

[89] Luan S, Reb J. Fast-and-frugal trees as noncompensatory models of performance-based personnel decisions. Organ Behav Hum Decis Process. 2017;141:29–42. doi: 10.1016/j.obhdp.2017.05.003

[90] Roessger KM. Attitudes Matter: Examining How Teaching Strategies for Attitudinal Change Help Adults Value Reflection and Calibrate Their Reflective Thinking. Adult Education Quarterly. 2023;73(3):266–85. doi: 10.1177/07417136231165007

[91] Edelson SA, Lo KD, Nelson T, Stark G, Stratton MT, van Esch C. From the shadow of overconfidence into the light of humility: Reflections on experiential learning activities gone awry. J Manag Educ. 2019;43(2):200–11. doi: 10.1177/1052562918812169

[92] Fust AP, Jenert T, Winkler C. Experiential or self-regulated learning: a critical reflection of entrepreneurial learning processes. Entrep Res J. 2018;8(2):20170098. doi: 10.1515/erj-2017-0098

[93] Barber BM, Odean T. Boys will be boys: Gender, overconfidence, and common stock investment. Q J Econ. 2001;116(1):261–92. doi: 10.1162/003355301556400

[94] Gill MJ, Swann WB, Silvera DH. On the genesis of confidence. Journal of Personality and Social Psychology. 1998;75(5):1101–14. doi: 10.1037/0022-3514.75.5.1101

[95] Bodnaruk A, Simonov A. Do financial experts make better investment decisions?. Journal of Financial Intermediation. 2015;24(4):514–36. doi: 10.1016/j.jfi.2014.09.001

[96] Merkl-Davies DM, Brennan NM. A conceptual framework of impression management: new insights from psychology, sociology and critical perspectives. Account Bus Res. 2011;41(5):415–37. doi: 10.1080/00014788.2011.574222

[97] Simon HA. Making management decisions: The role of intuition and emotion. Acad Manag Perspect. 1987;1(1):57–64. doi: 10.5465/ame.1987.4275905

[98] Campbell JY. Financial decisions and markets: A course in asset pricing. Princeton, NJ: Princeton University Press. 2018.

[99] Hřebačka V. On the testing of adaptive markets hypothesis using rolling windows. Appl Econ Lett. 2024;:1–5. doi: 10.1080/13504851.2024.2389341

[100] Cialdini RB, Goldstein NJ. Social influence: compliance and conformity. Annu Rev Psychol. 2004;55:591–621. doi: 10.1146/annurev.psych.55.090902.142015 14744228

[101] Sabir SA, Mohammad HB, Shahar HBK. The Role of Overconfidence and Past Investment Experience in Herding Behaviour with a Moderating Effect of Financial Literacy: Evidence from Pakistan Stock Exchange. Asian Economic and Financial Review. 2019;9(4):480–90. doi: 10.18488/journal.aefr.2019.94.480.490

[102] Loang OK. Financial stability at risk: evidence from market overreaction and herding behaviour in developed and emerging markets. China Finance Rev Int. 2025;15(1):67–92. doi: 10.1108/CFRI-06-2024-0322

[103] Ferreruela S, Mallor T. Herding in the bad times: The 2008 and COVID-19 crises. North American Journal of Economics and Finance. 2021;58:101531. doi: 10.1016/j.najef.2021.101531

[104] Yalcin KC, Tatoglu E, Zaim S. Developing an instrument for measuring the effects of heuristics on investment decisions. K. 2016;45(7):1052–71. doi: 10.1108/k-05-2015-0130

