# Supplementary material for: Adaptability factors and behavioral biases of investors in frontier markets: An adaptive market hypothesis perspective
Source: PLoS One. 2026 Mar 26;21(3):e0345883. doi: 10.1371/journal.pone.0345883 (PMC13020831; doi:10.1371/journal.pone.0345883)
Supplement: S1 Table — (DOCX) [file pone.0345883.s002.docx]

**Table 1. Socio-Demographic Profile of the Investors**

| **Variable** | **Category** | **Frequency** | **Percentage (%)** |
| --- | --- | --- | --- |
| Gender | Male | 480 | 75.0 |
|  | Female | 160 | 25.0 |
| Marital Status | Married | 483 | 75.5 |
|  | Unmarried | 152 | 23.8 |
|  | Divorced | 5 | 0.8 |
| Age (in years) | Less than 25 years | 46 | 7.2 |
|  | 25-34 years | 203 | 31.7 |
|  | 35-44 years | 244 | 38.1 |
|  | 45-54 years | 99 | 15.5 |
|  | 55–64 years | 35 | 5.5 |
|  | 65 years and above | 13 | 2.0 |
| Education level | High School/SSC/OL | 12 | 1.9 |
|  | Senior High School/HSC/AL | 25 | 3.9 |
|  | Bachelor’s degree | 194 | 30.3 |
|  | Master’s degree | 351 | 54.8 |
|  | Professional Course | 53 | 8.3 |
|  | Doctorate | 5 | 0.8 |
| Occupation | Private sector employee | 422 | 65.9 |
|  | Public sector employee | 54 | 8.4 |
|  | Retired | 35 | 5.5 |
|  | Self-employed | 95 | 14.8 |
|  | Unemployed | 34 | 5.3 |
| Monthly Income | Up to BDT 50,000 | 264 | 41.3 |
|  | BDT 50,001-1,00,000 | 195 | 30.5 |
|  | BDT 1,00,001-1,50,000 | 76 | 11.9 |
|  | 1,50,001 - 2,00,000 | 47 | 7.3 |
|  | 2,00,001 and above | 58 | 9.1 |
| State (Region) | Barishal | 43 | 6.7 |
|  | Chattogram | 117 | 18.3 |
|  | Dhaka | 357 | 55.8 |
|  | Khulna | 39 | 6.1 |
|  | Mymensingh | 20 | 3.1 |
|  | Rajshahi | 31 | 4.8 |
|  | Rangpur | 20 | 3.1 |
|  | Sylhet | 13 | 2.0 |
| Note: All amounts are reported in Bangladeshi Taka (BDT). | | | |

**Source(s):** Authors’ Own Creation
