# Supplementary material for: Adaptability factors and behavioral biases of investors in frontier markets: An adaptive market hypothesis perspective
Source: PLoS One. 2026 Mar 26;21(3):e0345883. doi: 10.1371/journal.pone.0345883 (PMC13020831; doi:10.1371/journal.pone.0345883)
Supplement: S2 Table — (DOCX) [file pone.0345883.s003.docx]

**Table 2. Measurement Model Results for Convergent Validity and Discriminant Validity**

| Convergent Validity | | | | Discriminant Validity |
| --- | --- | --- | --- | --- |
| Constructs | **Cronbach’s Alpha** | **CR** | **AVE** | **(HTMT < 0.85)** |
| ADV | 0.866 | 0.904 | 0.654 | Yes |
| DL | 0.789 | 0.855 | 0.543 | Yes |
| FL | N/A | N/A | N/A | Yes |
| EXP | N/A | N/A | N/A | Yes |
| HRD | 0.584 | 0.766 | 0.541 | Yes |
| MED | 0.732 | 0.827 | 0.552 | Yes |
| OVR | 0.821 | 0.87 | 0.527 | Yes |
| RFL | 0.721 | 0.823 | 0.541 | Yes |
| SCL | 0.702 | 0.816 | 0.527 | Yes |

**Source:** Authors’ Own Creation
