# Supplementary material for: Adaptability factors and behavioral biases of investors in frontier markets: An adaptive market hypothesis perspective
Source: PLoS One. 2026 Mar 26;21(3):e0345883. doi: 10.1371/journal.pone.0345883 (PMC13020831; doi:10.1371/journal.pone.0345883)
Supplement: S3 Table — (DOCX) [file pone.0345883.s004.docx]

**Table 3. Hypothesis Testing of Adaptability Factors Predicting Herding and Overconfidence Biases**

| Hypothesis | Path | Std. Beta (β) | p-value | 95% BC Confidence Interval | Decision |
| --- | --- | --- | --- | --- | --- |
| Herding Bias | | | | | |
| H1A | EXP → HRD | -0.047 | 0.114 | -0.110, 0.016 | Not Supported |
| H2A | RFL → HRD | -0.010 | 0.423 | -0.095, 0.076 | Not Supported |
| H3A | DL → HRD | -0.059 | 0.085 | -0.129, 0.012 | Not Supported |
| H4A | FL → HRD | -0.141 | <0.001 | -0.197, -0.084 | **Supported** |
| H5A | SCL → HRD | 0.309 | <0.001 | 0.240, 0.368 | **Supported** |
| H6A | ADV → HRD | 0.089 | 0.010 | 0.022, 0.150 | **Supported** |
| H7A | MED → HRD | 0.194 | <0.001 | 0.124, 0.260 | **Supported** |
| Overconfidence Bias | | | | | |
| H1B | EXP → OVR | 0.261 | <0.001 | 0.192, 0.328 | **Supported** |
| H2B | RFL → OVR | 0.113 | 0.024 | 0.015, 0.203 | **Supported** |
| H3B | DL → OVR | 0.258 | <0.001 | 0.181, 0.326 | **Supported** |
| H4B | FL → OVR | -0.067 | 0.038 | -0.126, -0.003 | **Supported** |
| H5B | SCL → OVR | 0.056 | 0.103 | -0.015, 0.131 | Not Supported |
| H6B | ADV → OVR | 0.074 | 0.067 | -0.009, 0.154 | Not Supported |
| H7B | MED → OVR | 0.009 | 0.423 | -0.069, 0.084 | Not Supported |

**Source(s):** Authors’ Own Creation

**Notes:**

- BC Confidence Interval = Bias-Corrected 95% Confidence Interval.

Bolded hypotheses indicate statistically supported relationships (p < 0.05, one-tailed).
