# Supplementary material for: Adaptability factors and behavioral biases of investors in frontier markets: An adaptive market hypothesis perspective
Source: PLoS One. 2026 Mar 26;21(3):e0345883. doi: 10.1371/journal.pone.0345883 (PMC13020831; doi:10.1371/journal.pone.0345883)
Supplement: S3 Text — Non-parametric bootstrapping (10,000 resamples) was applied to generate stable standard errors and 95% bias-corrected confidence intervals for all structural paths. Model fit indices included SRMR (saturated = 0.072; estimated = 0.073), d_ULS (2.946; 3.007), and d_G (0.606; 0.608), supporting overall adequacy. The model showed moderate explanatory power (R2 = 0.225 for herding; 0.189 for overconfidence) and positive Q2 values, indicating predictive relevance. VIF (< 5) confirmed absence of multicollinearity, while residual diagnostics supported linearity and homoscedasticity. Durbin-Watson statistics further indicated no autocorrelation of errors. These extended diagnostics reinforce that the model was robust, well specified, and theoretically meaningful despite reliance on purposive sampling. (DOCX) [file pone.0345883.s005.docx]

**S3 Text. Extended Robustness and Model Diagnostics**

Non-parametric bootstrapping (10,000 resamples) was applied to generate stable standard errors and 95% bias-corrected confidence intervals for all structural paths. Model fit indices included SRMR (saturated = 0.072; estimated = 0.073), d_ULS (2.946; 3.007), and d_G (0.606; 0.608), supporting overall adequacy. The model showed moderate explanatory power (R² = 0.225 for herding; 0.189 for overconfidence) and positive Q² values, indicating predictive relevance. VIF (< 5) confirmed absence of multicollinearity, while residual diagnostics supported linearity and homoscedasticity. Durbin-Watson statistics further indicated no autocorrelation of errors. These extended diagnostics reinforce that the model was robust, well specified, and theoretically meaningful despite reliance on purposive sampling.
