# Supplementary material for: Adaptability factors and behavioral biases of investors in frontier markets: An adaptive market hypothesis perspective
Source: PLoS One. 2026 Mar 26;21(3):e0345883. doi: 10.1371/journal.pone.0345883 (PMC13020831; doi:10.1371/journal.pone.0345883)
Supplement: S4 Table — (DOCX) [file pone.0345883.s006.docx]

**Table 4. PLS-SEM Model Fit and Predictive Diagnostics**

| **Index** | **Saturated Model** | **Estimated Model** | **Notes / Thresholds** |
| --- | --- | --- | --- |
| SRMR | 0.072 | 0.073 | <0.08 acceptable |
| d_ULS | 2.946 | 3.007 | Comparative only |
| d_G | 0.606 | 0.608 | Comparative only |
| R² (Herding) | 0.225 | – | Moderate explanatory power |
| R² (Overconfidence) | 0.189 | – | Moderate explanatory power |
| Q² (Herding) | > 0 | – | Predictive relevance confirmed |
| Q² (Overconfidence) | > 0 | – | Predictive relevance confirmed |
| VIF (all constructs) | < 5 | – | No multicollinearity |
| Durbin-Watson | ~2.0 | – | No autocorrelation of errors |
| Residual plots | – | – | Linearity, homoscedasticity ok |

**Source:** Authors’ Own Creation
