# Supplementary material for: Adaptability factors and behavioral biases of investors in frontier markets: An adaptive market hypothesis perspective
Source: PLoS One. 2026 Mar 26;21(3):e0345883. doi: 10.1371/journal.pone.0345883 (PMC13020831; doi:10.1371/journal.pone.0345883)
Supplement: S5 Table — (DOCX) [file pone.0345883.s007.docx]

**Table 5. Comparative Dynamics of Adaptability Factors and Behavioral Biases Across Frontier, Emerging, and Developed Markets**

| Market Type | Key Adaptability Drivers | Impact on Herding | Impact on Overconfidence |
| --- | --- | --- | --- |
| Frontier (Bangladesh, Sri Lanka, Pakistan) | External (SCL, ADV, MED); Internal (EXP, RFL, DL) | Strongly driven by external factors | Internal factors amplify OVR |
| Emerging (Kenya, India, Saudi Arabia) | Internal dominant (EXP, RFL); External less impactful | Mixed; HRD less externally driven | Internal factors raise OVR; FL moderates |
| Developed (Spain, Portugal, G7 countries) | High systemic transparency | Low HRD due to regulatory clarity | Low OVR via low trade volume and return dynamics |

**Source(s):** Authors’ Own Compilation
