# Supplementary figures and images for: Adaptability factors and behavioral biases of investors in frontier markets: An adaptive market hypothesis perspective
Source: PLoS One. 2026 Mar 26;21(3):e0345883. doi: 10.1371/journal.pone.0345883 (PMC13020831; doi:10.1371/journal.pone.0345883)

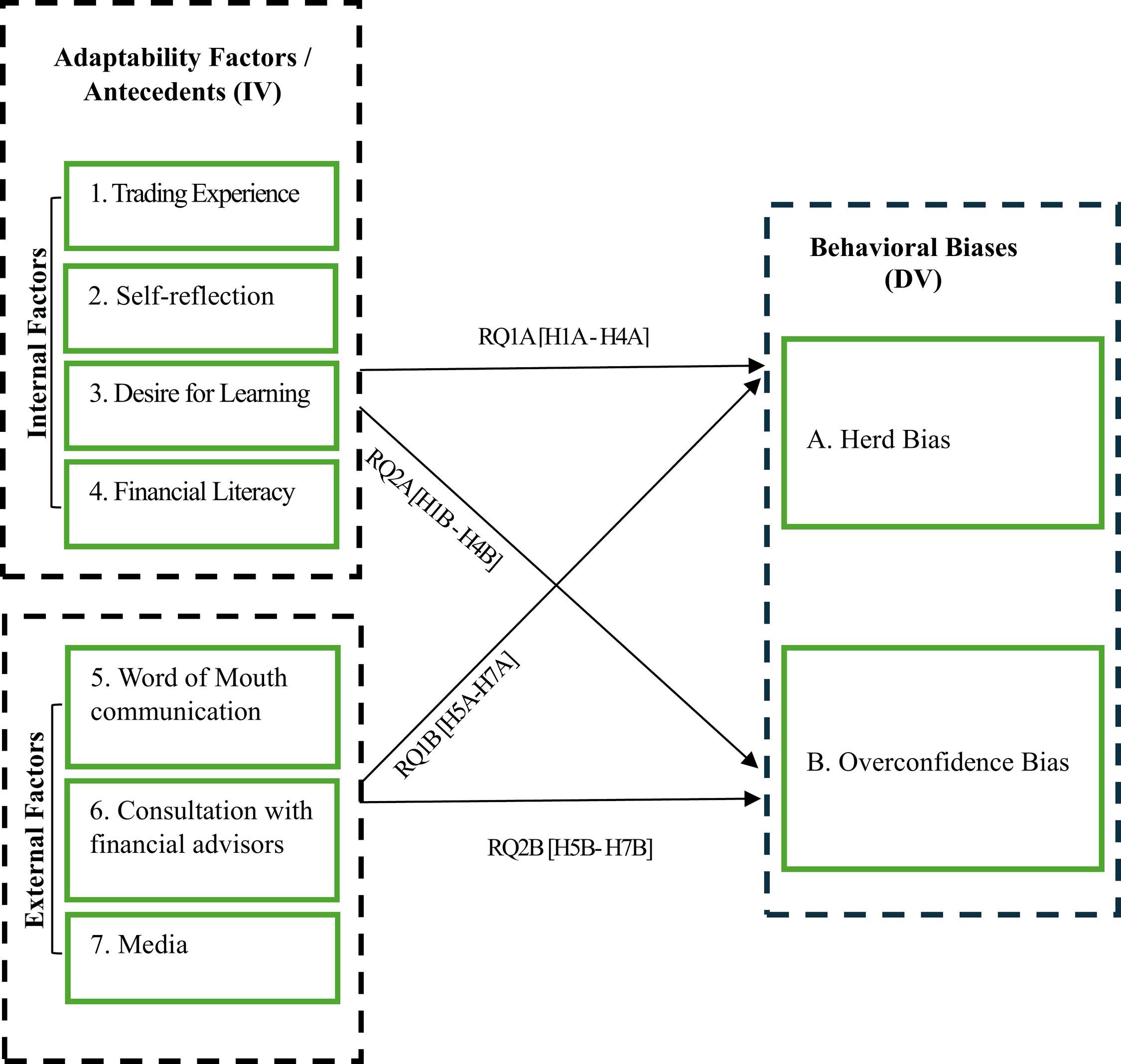

Supplement: S1 Fig — (TIF) [file pone.0345883.s008.tif]

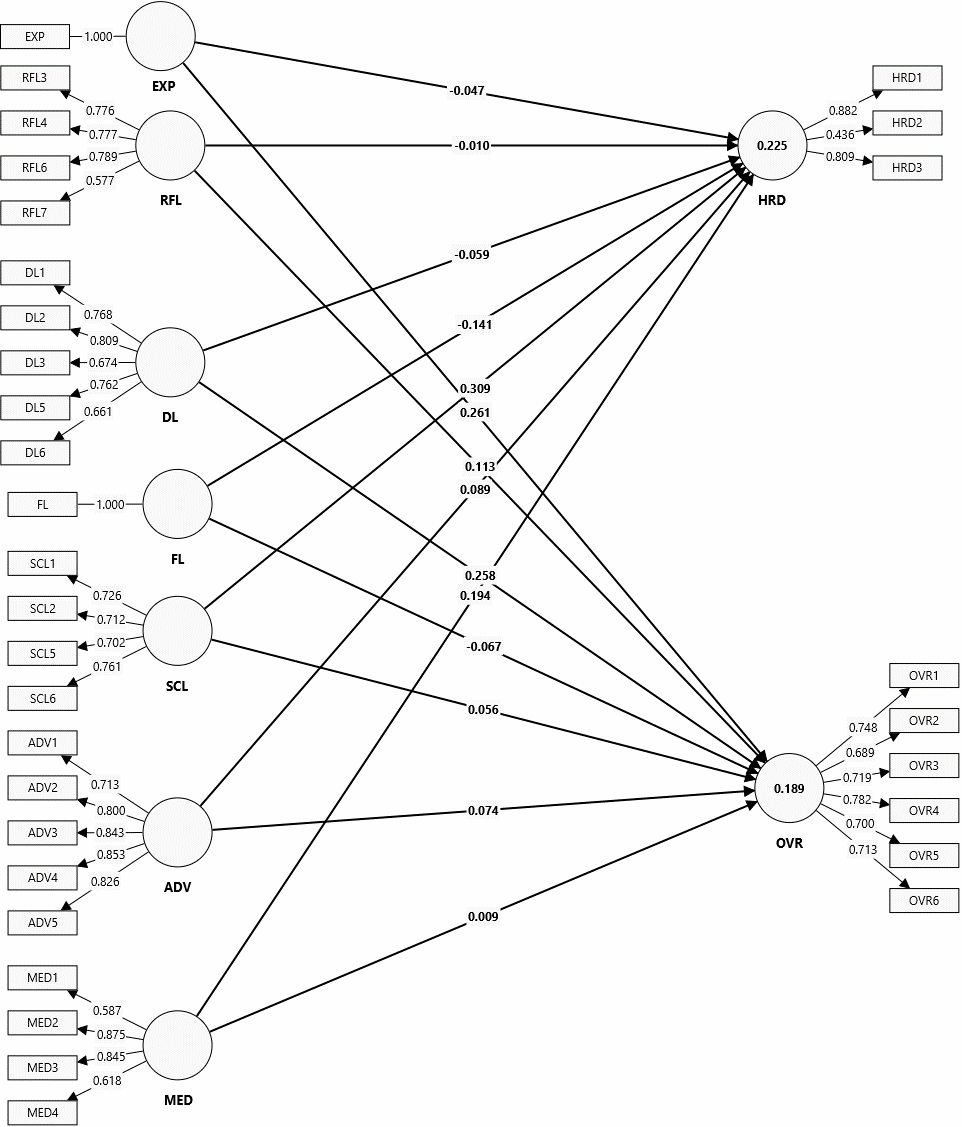

Supplement: S2 Fig — (TIF) [file pone.0345883.s009.tif]
